# An Eluate of the Medicinal Plant *Garcinia kola* Displays Strong Antidiabetic and Neuroprotective Properties in Streptozotocin-Induced Diabetic Mice

**DOI:** 10.1155/2022/8708961

**Published:** 2022-03-21

**Authors:** Paul F. Seke Etet, Muaawia A. Hamza, Ahmed El-Tahir, Lorella Vecchio, Sayed Y. Osman, Gwiria M. H. Satti, Mohamed H. A. Ismail, Mohammed Farahna, Alfred K. Njamnshi, Abdu Adem

**Affiliations:** ^1^Department of Physiological Sciences and Biochemistry, Faculty of Medicine and Biomedical Sciences, University of Garoua, Garoua, Cameroon; ^2^Center for Sustainable Health and Development, Garoua, Cameroon; ^3^Neuroscience Laboratory, Faculty of Medicine and Biomedical Sciences, The University of Yaounde I & Brain Research Africa Initiative (BRAIN), Yaounde, Cameroon; ^4^Faculty of Medicine, King Fahad Medical City, MOH, Riyadh, Saudi Arabia; ^5^Research Centre, King Fahad Medical City, MOH, Riyadh, Saudi Arabia; ^6^Faculty of Medicine, Nile University, Khartoum, Sudan; ^7^Department of Biochemistry and Molecular Biology, Al-Neelain University, Khartoum, Sudan; ^8^Department of Biochemistry, Faculty of Medicine, University of Khartoum, Khartoum, Sudan; ^9^Biochemistry Department, Faculty of Science, King Saud University, Riyadh, Saudi Arabia; ^10^Development & Innovation Centre, Tabil Factory for Food Industries, Kafori, Khartoum, Sudan; ^11^Department of Pharmacology and Therapeutics, College of Medicine and Health Sciences, Khalifa University, Abu Dhabi, UAE

## Abstract

**Materials and Methods:**

*G. kola* methanolic extract was fractionated using increasingly polar solvents. Fractions were administered to streptozotocin (STZ)-induced diabetic mice until marked motor signs developed in diabetic controls. Fine motor skills indicators were measured in the horizontal grid test (HGT) to confirm the prevention of motor disorders in treated animals. Column chromatography was used to separate the most active fraction, and subfractions were tested in turn in the HGT. Gas chromatography-mass spectrometry (GC-MS) technique was used to assess the components of the most active subfraction.

**Results:**

Treatment with ethyl acetate fraction and its fifth eluate (F5) preserved fine motor skills and improved the body weight and blood glucose level. At dose 1.71 mg/kg, F5 kept most parameters comparable to the nondiabetic vehicle group values. GC-MS chromatographic analysis of F5 revealed 36 compounds, the most abundantly expressed (41.8%) being the *β*-lactam molecules *N*-ethyl-2-carbethoxyazetidine (17.8%), *N*,*N*-dimethylethanolamine (15%), and isoniacinamide (9%).

**Conclusions:**

Our results suggest that subfraction F5 of *G. kola* extract prevented the development of motor signs and improved disease profile in an STZ-induced mouse model of diabetic encephalopathy. Antidiabetic activity of *β*-lactam molecules accounted at least partly for these effects.

## 1. Introduction

The incidence of type 1 diabetes mellitus (T1D) has been increasing worldwide [1, 2]. The hallmark of the disease is a metabolic syndrome with very high glycemia resulting from deficits in insulin production. T1D encompasses severe microvascular complications such as cognitive and motor dysfunctions, which are particularly common in pediatric populations and young adults [3–5]. Disorders such as early behavioral alterations, which predict later psychiatric morbidity in one-third of cases in patients [6–8], are common in streptozotocin (STZ)-injected murine models [9–11]. Evidence from epidemiological, clinical, and experimental studies suggests that these complications emerge mainly from inappropriate glycemic control, a puzzling issue also associated with several other complications of T1D, higher hospitalization costs, and higher mortality [12, 13]. These complications are fueled by sustained neuroinflammation encompassing mitochondrial dysfunction, excitotoxicity, gliosis, nervous tissue damage, and cell loss. Losses in inflammation-sensitive large neurons such as pyramidal cells, cerebellar nucleus neurons, and Purkinje cells result in marked deficits in motor coordination in both human and experimental diseases [14–17]. Thus, antidiabetic drugs with neuroprotective properties are highly needed in the clinical practice.

*Garcinia kola* Heckel (Clusiaceae/Guttiferae) is an African rain forest plant whose seeds, best known as “Onie” in Fang-Beti languages (Cameroon, Gabon, and Equatorial Guinea), “Orogbo” in Yoruba language (Nigeria), or bitter kola, all over West Africa, are eaten recreationally and used in traditional medicine to treat T1D and malaria [18–20]. Early reports suggested that *G. kola* seeds have anti-inflammatory, antidiabetic, and neuroprotective properties [21–23]. Considering that these studies were mainly performed in animals acutely or subchronically diabetic, we performed a study in long-term diabetic animals [24]. We observed that the oral administration of a suspension of *G. kola* seeds in distilled water or methanolic extract improved the gait and posture of STZ-induced diabetic rats, major indicators of motor function disturbance in this model and in the human disease. These effects were mediated partly by protecting cerebellar neurons from apoptosis and by decreasing neuroinflammation [24, 25]. Moreover, we recently reported the ability of *G. kola* to improve cognitive and motor functions in a rat model of acute radiation syndrome [26–27]. Interestingly, at the same time, other recent studies also reported *G. kola* neuroprotective activities in various models of neurodegenerative diseases and conditions [28–31], calling for further studies to develop neuroprotective drugs from *G. kola* seeds.

The aim of the present study was to perform a guided fractionation of *G. kola* methanolic extract based on the ability to prevent the development of motor signs in an STZ-induced mouse model of diabetic encephalopathy as a preclinical step for developing a new antidiabetic drug with neuroprotective properties. The phytoconstituents present in the most active subfraction isolated were assessed as well.

## 2. Materials and Methods

### 2.1. Animals

Healthy young adult male Swiss mice (6 months old, 24 ± 3.2 g, *N* = 86) were obtained from the College of Pharmacy, King Saud University (Riyadh, Saudi Arabia) and acclimatized to laboratory conditions. Insulin insufficiency was induced by injecting animals (60 mg/kg body weight, i.p.) once with STZ in citrate buffer (Sigma Aldrich, USA, pH = 4.5). Then, the experimental groups received daily (*per os*.): (i) the vehicle solution (DMSO) (diabetic control and nondiabetic control groups); (ii) insulin (0.4 UI/kg, s.c., Mixtard suspension, Novo Nordisk A/S, Zürich, Switzerland); or (iii) *G. kola* extracts. The animals were housed in groups of 3 or 4, kept at 23.4°C in a 12/12 h dark-light cycle, with *ad libitum* access to water and food and permanent video recording aimed at detecting adverse signs or marked behavioral changes. Considering that signs of nervous system involvement are present from the first month post-STZ injection in laboratory rodents [25], the treatment started two weeks after the STZ injection and lasted two weeks for each of the three phases of the present study (see Section 2.2) to avoid unnecessary suffering to the animals.

The procedures were approved by King Fahad Medical City (Riyadh, Saudi Arabia) (IRB log 16–343, IRB registration number with OHRP/NIH: IRB00010471). The European Union guidelines for the ethical use of animals in scientific research (Directive 2010/63/EU) were observed.

### 2.2. Experimental Procedures

Fine motor skill indicators measured in the horizontal grid test (HGT), such as the proportion of normal steps, the animal's posture, and the grasping ability of forepaws and hind paws [32–34], were used to evaluate cognitive function in control groups (*N* = 6 per group) and in diabetic mice treated with plant extracts (*N* = 5 per group) and fractions (*N* = 6 per group). In the first series of experiments, the effects of hexane, dichloromethane, ethyl acetate sequential fractions of *G. kola* extract, and of the remainder (termed as water fraction) were assessed. The mice received doses of fractions equivalent to twice the content in the crude extract's effective dose (100 mg/kg), whose antidiabetic properties were previously reported in other models of diabetic encephalopathy [24, 25]. Afterwards, the most active fraction of *G. kola* was separated using column chromatography.

In the second series of experiments, other diabetic animals were treated with eluates (“subfractions”), and their fine motor skills were evaluated similarly. Then, the secondary metabolites present in the most active subfraction were determined using the gas chromatography-mass spectrometry (GC-MS) technique. In the third series of experiments, other diabetic animals treated with dilutions of the most active subfraction (1/2, 1/4, 1/10, and 1/20) were tested in the HGT to assess the dose-response profile of the effects observed. The cognitive function improvement mediated by this subfraction was further evaluated in two other behavioral tests: the open-field test (OFT) and the hole-board test (HBT).

During the study, each animal's weight was measured every 3 days and the blood sugar level was measured weekly. The animals were sacrificed under deep anesthesia at the end of each series of experiments. Brains were dissected out and processed for (i) histopathological studies assessing nervous tissue damage and neuronal loss (hematoxylin and eosin/H&E staining) and (ii) immunohistochemical studies assessing the expressions of markers of neuroinflammation and neurodegeneration.

### 2.3. Plant Material Processing

#### 2.3.1. Extraction

Fresh *G. kola* seeds were purchased from West African sellers in Central Saudi Arabia. The seeds were authenticated at Qassim University College of Agriculture, and specimens (Voucher No 2016_02334) were stored. The seeds were dried in the shade at laboratory temperature (25–28°C), peeled, sliced, ground with an electrical mill, and further pulverized with an electric blender. Then, the powdered material (600 g) was macerated in 80% methanol for 72 h. After filtration (Whatman filter paper No. 2) and methanol evaporation (rotary evaporator, 40°C), the extract was kept at 4C°. The extraction yield was 25.1%.

#### 2.3.2. Fractionation and Chromatographic Separation of the Extract

The extract fractionation and chromatographic separation were performed using standard procedures [35, 36]. Briefly, the extract was suspended in distilled water, fractionated sequentially with *n*-hexane, dichloromethane, and ethyl acetate, and each subextract (fraction) was dried (rotary evaporation, 40°C). Then, the remainder (water fraction) was freeze-dried. The extraction yields were 2.6% (hexane), 14.5% (dichloromethane), 4.1% (ethyl acetate), and 6.5% (water). The doses of fractions administered included approximately twice the amounts in the effective dose of crude extract (100 mg/kg) [24, 25], i.e., 4.5 mg/kg, 28.7 mg/kg, 7.7 mg/kg, and 12.9 mg/kg for hexane, dichloromethane, ethyl acetate, and water fractions, respectively.

Ethyl acetate fraction, which had the strongest motor skills' improvement effect (see Section 3), was processed for chromatographic separation with a 60 mm × 16 mm column filled with Sephadex LH-20 (GE Healthcare, Chicago, Illinois, USA). Briefly, a mass of 4.09 g of the ethyl acetate fraction was dissolved in 5 mL of HPLC grade methanol, and the extract was eluted with a flow rate of 1 mL/min. Based on the elution order and the UV absorbance, elutes were pooled into subfractions called fractions F1–F5. The subfractions were evaporated under reduced pressure and stored at −20°C. The extraction yields were 0.03%, 0.13%, 0.04%, 0.42%, and 0.69% for F1–F5, respectively. The doses of subfractions administered included approximately 10 times the amounts in dose 100 mg/kg of crude extract, i.e.: 0.33 mg/kg, 1.26 mg/kg, 0.4 mg/kg, 4.16 mg/kg, and 6.85 mg/kg for F1–F5, respectively.

#### 2.3.3. Identification of Components of the Subfraction F5

As F5 appeared to have the strongest motor skills' improvement effect (see Section 3), this subfraction was processed for phytoconstituent detection with GC-MS technique, using an Agilent 6890 gas chromatograph coupled to a 5973 Mass Selective Detector with helium as carrier gas and a DB-5MS (Agilent) fused silica capillary column [35, 36]. The gas chromatography was temperature programmed (from 65°C, 2 min initial time, to 310°C at 6°C min^−1^, isothermal for 55 min final time). The mass spectrometry was operated in the electron impact mode (70 eV ion source energy) and the GC–MS ChemStation data system was used for obtaining and processing mass spectrometric data. Phytoconstituents were identified by comparison with mass spectra and chromatographic retention characteristics of the mass spectral library of the GC-MS data system.

### 2.4. Assessment of Motor and Cognitive Functions: Behavioral Tests

#### 2.4.1. HGT

The HGT (inverted screen test) is a well-established test for combined forepaw and hindpaw strength evaluation [33, 34]. The test was started by placing mice on a wire grid inverted afterward over a foam pad and ended when the animal fell off the grid or after 2 min. The animal performance on the grid was video-recorded. Changes in the animal's posture and in the grasping ability of forepaws and hind paws were analyzed and scored offline. Good steps, defined as steps without paw tremor and efficiently reaching their target, were counted and expressed as a percentage of the total number of steps on the grid.

#### 2.4.2. OFT

The open-field arena was a 38.1 cm high transparent Plexiglas box with a 40.6 cm × 40.6 cm floor including a 20.2 cm × 20.2 cm central zone and a peripheral zone [37, 38]. At the start of the test, a mouse was placed facing the wall at an angle of the arena and the activity of the animal was video-recorded for 10 min. The camera was placed 50 cm above the arena with a 45° angle for simultaneous capture of vertical and horizontal activities. After each trial, the floor and walls of the arena were cleaned with 70% alcohol solution. The number of entries and time spent in the central zone and the distance traveled in the arena were determined with the Image Processing Toolbox® of MATLAB software (MathWorks, Natick, MA), using motion tracking on image sequences. The episodes of freezing (more than 3 sec immobility with characteristic posture), rearing, and grooming were scored from video recordings.

#### 2.4.3. HBT

The hole-board arena was a 40.6 cm × 40.6 cm × 60 cm transparent Plexiglas box with 16 equidistant holes (2.5 cm in diameter) cut into the floor [37, 39]. The test was started by placing a mouse in the corner of the arena, facing the wall. The animal vertical and horizontal activities were video-recorded for 10 min, using a computerized digital camera placed 50 cm above the arena (45° angle). After each trial, the floor and walls of the arena were cleaned with 70% alcohol solution. Tests were performed under bright white lighting produced by fluorescent lamps (∼500 lux vs. ∼300–400 lux at home cage floor). The latency to the first head dipping and the head dipping number and time was determined from video recordings.

### 2.5. Tissue Processing and Staining

At sacrifice, the animals were sequentially perfused with phosphate buffer saline (pH 7.4) and Karnovsky's fixative (5% Glutaraldehyde, 4% formaldehyde in 0.08 M buffer) under deep anesthesia. Brains were dissected out, postfixed for 2 h in Karnovsky's fixative, then processed for paraffin embedding using a tissue processor. Embedded brains were cut entirely in the transversal plane (thickness 5 *µ*m). Sections were mounted subsequently on six different slides to obtain a 30 *µ*m distance between adjacent sections on the same slide. A series of sections were processed for H&E staining using the standard protocol. Histopathological analyses assessing signs of tissue damage were performed throughout the brain, with a special focus on large neurons of the cerebellum (Purkinje cells and deep nucleus cells) and of the motor cortex (pyramidal neurons). The analyses were performed using a computerized light microscope, under 20x, 40x, and 120x objectives.

### 2.6. Immunohistochemistry

Four series of sections were deparaffinized in xylene and rehydrated. Endogenous peroxidase activity was extinguished with 10% H_2_O_2_ and heat-induced antigen retrieval was performed using Tris-EDTA buffer (1 mM EDTA solution, 10 mM Tris base, and 0.05% Tween 20 in distilled water, pH 9). Sections were incubated overnight in a buffer solution (5% skim milk, 0.1% Tween 20 in Tris-buffered saline) containing one of the following primary antibodies: rabbit anti-TNF-*α*, rabbit anti-iba1, goat anti-caspase 3, and goat anti-Fas (1 : 100, Santa Cruz Biotechnology, CA). Then, sections were incubated in an HRP-conjugated secondary antibody and processed according to the instructions of the kit manufacturer (ABCAM, Cambridge, UK). Finally, they were stained with chromogen substrate 3,3′-diaminobenzidine hydrochloride (DAB, ABCAM) (10 min), counterstained with hematoxylin (ABCAM) (5 min), dehydrated through a graded ethanol series, cleared in xylene, and covered with a glass coverslip. Tris-TBS buffer (0.1% Tween 20 in Tris-buffered saline, pH = 7.6) was used for interstep rinsing, as recommended by the kit manufacturer (ABCAM). Expressions of these markers of inflammation (Tumor Necrosis Factor-alpha/TNF-*α*, iba1), inflammation-related cell death (Fas receptor), and apoptosis (caspase-3) were observed using a light microscope under 20x, 40x, and 120x objectives.

### 2.7. Data Analysis

Body weight, blood sugar level, and performance on behavioral tests of diabetic animals treated with fractions of *G. kola* extract, subfractions of ethyl acetate fraction (F1 to F5), and lower doses of F5 were compared to diabetic control, insulin group, and nondiabetic vehicle group. Comparisons were made using two-way ANOVA and LSD post hoc test (OriginPro 8® software version 9.75, OriginLab Corporation, Northampton, MA). Differences with *P* < 0.05 were considered significant. Data were presented as mean ± SEM.

## 3. Results

### 3.1. Clinicopathological Observations

**Figure 1:**
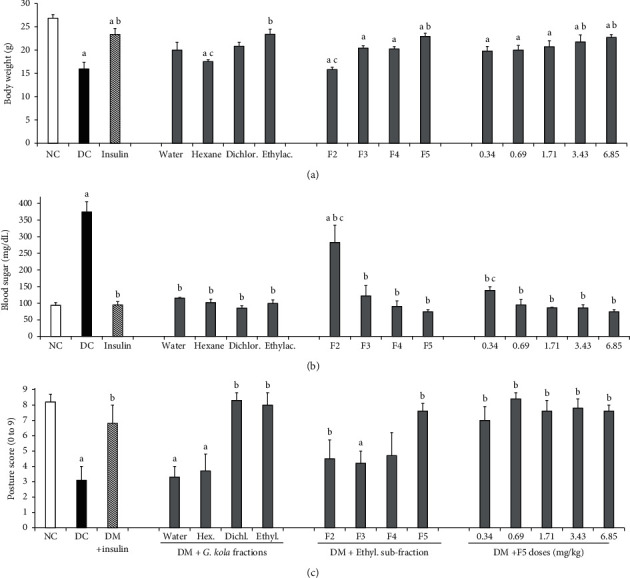
Body weight, blood sugar and posture. Effects of treatment with *G. kola* extracts and fractions on the body weight (a), the blood sugar level (b), and the animal's posture score (c). The marked decrease in body weight (a), increase in blood sugar level (b), and decrease in the animal's posture score (c) in diabetic control animals (DC) were mitigated by treatment with insulin and most *G. kola* fractions. ANOVA + LSD test: ^a^*P* < 0.05 vs. nondiabetic control (NC) group; ^b^*P* < 0.05 vs. DC group; ^c^*P* < 0.05 vs. insulin group. Data are mean ± SEM. *N* = 6 for NC and DC groups. *N* = 5 for insulin and *G. kola* test groups.

Indicators of central nervous system functional disturbances were not observed in nondiabetic animals following vehicle administration. Their body weight normally increased throughout the study (Table 1 and Figure 1(a)) and their blood sugar level remained normal (Figure 1(b)). On the other hand, marked decreases in body weight (Table 1) and increases in blood sugar level (Figure 1(b)) were observed in diabetic controls and in all diabetic animals before treatment. The severity of these alterations increased with time and became significant from week 2 post-STZ injection (Table 1). Two weeks after STZ injection, diabetic control group animals displayed increasingly marked disease signs and indicators of nervous system involvement, such as (i) indicators of severe systemic disease like cachexia, porphyrin deposits around the eyes, and shaggy fur; (ii) indicators of pain like increased vocalization at handling; (iii) indicators of depression-like mood disorders such as freezing behavior and decreased social interactions; (iv) indicators of motor impairment like poor posture, ataxia, and other gait disturbances.

### 3.2. Body Weight and Blood Sugar Level

#### 3.2.1. Effects of *G. kola* Extract Fractions

Significant increase in blood sugar level and decrease in body weight were observed in the diabetic control group compared to the nondiabetic vehicle group (*P*=0.0019 and *P* < 0.0001, respectively) (Table 1 and Figures 1(a) and 1(b)). Blood sugar level was decreased compared to diabetic control group in animals treated with the water fraction of *G. kola* methanolic extract (*P*=0.004), hexane fraction (*P*=0.0017), dichloromethane fraction (*P*=0.0018), and ethyl acetate fraction (*P*=0.0015), or with insulin (*P*=0.0014) (Figure 1(b)). The body weight was increased (improved) in diabetic animals treated with all fractions of the methanolic extract (*P* < 0.001), except for the water fraction (*P*=0.746) (Table 1). The bodyweight improvement mediated by *G. kola* extract was more marked in the second week of treatment (Table 1, Figure 1(a)). As expected, insulin treatment decreased the blood sugar level and improved the body weight of diabetic animals (*P*=0.0014 and *P*=0.0002 vs. diabetic control group, respectively) (Table 1 and Figures 1(a) and 1(b)). However, although treatment with insulin and *G. kola* extract's fractions improved blood sugar level to values close to normal (i.e., no significant difference compared to the nondiabetic vehicle group) (Figure 1(b)), none of these treatments restored the body weight to the nondiabetic vehicle group values (*P* < 0.01 vs. nondiabetic vehicle group) (Table 1 and Figure 1(a)). Animals treated with ethyl acetate fraction regained weight faster than those treated with other fractions (Table 1). Just like insulin, the ethyl acetate fraction raised the body weight beyond the baseline value (body weight at the beginning of the experiment) (Table 1).

Furthermore, dichloromethane and ethyl acetate fractions, but not water and hexane fractions, improved animal appearance and other disease signs. Considering these observations and effects on fine motor skill indicators (see Section 3.3), the ethyl acetate fraction was separated further, and the subfractions were tested. Insulin also improved disease signs and animal appearance.

#### 3.2.2. Effects of Ethyl Acetate Subfractions

The effects of ethyl acetate subfractions on body weight and blood sugar level are shown in Table 1 and Figures 1(a) and 1(b), respectively. Ethyl acetate subfraction 1 (F1) induced a marked decrease in blood sugar level and an accelerated decrease in body weight, resulting in the death of all animals in this test group in the first 3 days of treatment (data not shown). On the other hand, subfractions F2–F5 improved the blood sugar concentration to levels comparable to the nondiabetic vehicle group and insulin group (as indicated by no significant difference from these groups) (Figure 1(b)). The blood sugar levels in groups treated with subfractions F2–F5 and insulin were significantly lower than the diabetic control group (*P*=0.0017, *P*=0.0012, *P*=0.0004, *P*=0.0013, and *P*=0.0011, respectively) (Figure 1(b)).

Of all fractions and subfractions, only F5 improved body weight beyond baseline values at the first week of treatment (*P*=0.002 vs. diabetic control group) (Table 1 and Figure 1(a)). F5 restored body weight growth to levels comparable to the nondiabetic vehicle group in the second week of treatment (Table 1). These effects were stronger than insulin treatment (8.4% more in the first week and 23.2% more in the second week, *P*=0.035) that failed to restore body weight growth to the nondiabetic vehicle group values (Table 1 and Figure 1(a)). F5 also improved disease signs better than other subfractions and previous fractions tested. However, animals treated with F5 displayed signs of aggressiveness that were not observed in the nondiabetic vehicle group, in the diabetic controls, and in animals treated with other subfractions. We hypothesized that such effects may emerge from neurotoxicity associated with too high doses. Thus, lower doses of F5 were tested to assess the dose-response profile of this subfraction. The doses tested were: 3.43, 1.71, 0.69, and 0.34 mg/kg, corresponding to 1/2, 1/4, 1/10, and 1/20 dilutions of F5.

For body weight and blood sugar level, results of the assessment of the dose-response profile of F5 are shown in Table 1 and Figures 1(a) and 1(b). F5 increased the body weight in a dose-dependent fashion, with effects comparable to or stronger than insulin at the dose 1.71 mg/kg (*P* < 0.05) (Table 1 and Figure 1(a)). The blood sugar level was also improved in a dose-dependent fashion, with significant effects from the dose 0.69 mg/kg (*P* < 0.001 in the second week of treatment) (Figure 1(b)). Doses higher than 0.34 mg/kg markedly improved the diabetic animal's condition and appearance, and no sign of aggressiveness was observed in doses lower than 3.43 mg/kg.

### 3.3. Animal's Posture and Fine Motor Skill Indicators in HGT Test

Diabetic controls displayed a marked decrease in the animal's posture score (*P*=0.005 vs. nondiabetic control group), which was improved by treatments with insulin (*P*=0.0009 vs. diabetic control group), dichloromethane, and ethyl acetate fractions of *G. kola* extract (*P*=0.0009 and *P*=0.0056 vs. diabetic control group, respectively), as well as F2 and F5 subfractions of ethyl acetate fraction (*P*=0.0067 and *P*=0.005 vs. diabetic control group, respectively) (Figure 1(c)). F5 effects grew with the dose-administered, with the strongest effects around dose 0.69 mg/kg (*P*=0.0002 vs. diabetic control group) (Figure 1(c)).

**Figure 2:**
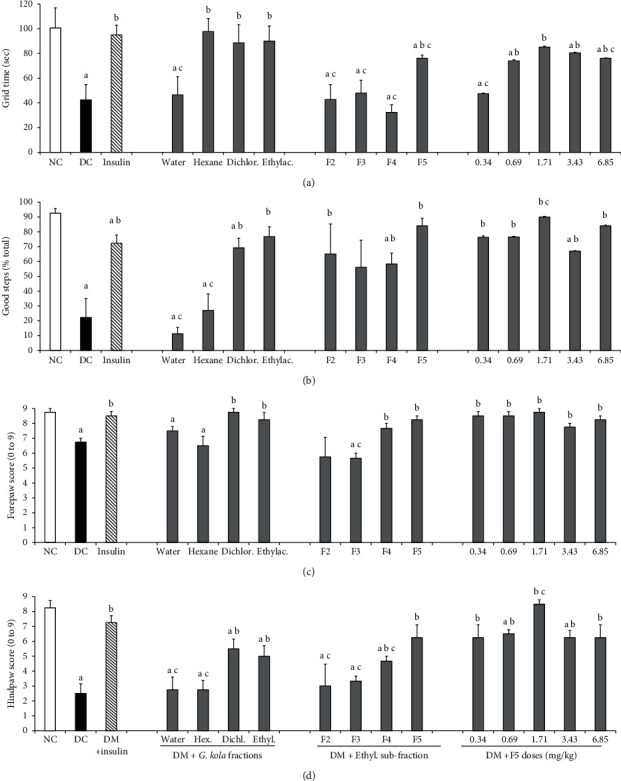
Grid test motor skill indicators. Effects of *G. kola* extracts and fractions on horizontal grid test's fine motor skill indicators: the time spent on the grid (a), the relative number of good steps (b), and forepaw (c) and hindpaw (d) scores. Note that the marked decreases observed in diabetic controls were mitigated by treatment with insulin and most *G. kola* extracts and fractions. ANOVA + LSD test: ^a^*P* < 0.05 vs. nondiabetic controls (NC) group; ^b^*P* < 0.05 vs. diabetic control (DC) group; ^c^*P* < 0.05 vs. insulin group. Data are mean ± SEM. *N* = 6 for NC and DC groups. *N* = 5 for insulin and test groups.

The effects of *G. kola* fractions on indicators of fine motor skills and other motor functions revealed by the HGT test are shown in Figure 2. The time spent on the horizontal grid was decreased in the diabetic control group compared to the nondiabetic vehicle group (*P*=0.007). Just as insulin (*P*=0.016), hexane, dichloromethane, and ethyl acetate fractions of the extract of *G. kola* seeds prevented this decrease (*P*=0.015, *P*=0.04, and *P*=0.035 vs. diabetic control group, respectively) (Figure 2(a)). In the study of subfraction effects, only the F5 and its sub-doses equal or higher than 0.69 mg/kg prevented decreases in the time spent on the horizontal grid (*P* < 0.01 vs. diabetic control group) (Figure 2(a)). However, F5 and its subdoses failed to prevent a decrease in the time spent on the horizontal grid (*P* < 0.05 vs. nondiabetic vehicle group) (Figure 2(a)), except for dose 1.71 mg/kg whose effects were comparable to both nondiabetic vehicle and insulin-treated groups (no significant difference between these groups and the dose of F5) (Figure 2(a)).

The percentage of good steps (efficient and without tremor) on the grid was significantly decreased in the diabetic control group (*P*=0.008 vs. nondiabetic vehicle group). *G. kola* dichloromethane and ethyl acetate fractions and insulin prevented this decrease (*P*=0.026, *P*=0.015, *P*=0.022 vs. diabetic control group, respectively) (Figure 2(b)). The decrease in good step percentage was also improved by ethyl acetate subfractions F2–F5 (*P* < 0.05 vs. diabetic control group) (Figure 2(b)). However, unlike insulin (*P* < 0.026 vs. nondiabetic vehicle group), treatment with F5 dose 1.71 mg/kg kept the percentage of good steps at nondiabetic vehicle group values (*P*=0.109 vs. nondiabetic vehicle group) (Figure 2(b)).

The forepaw performance score was significantly decreased in the diabetic control group (*P*=0.001 vs. nondiabetic vehicle group). This decrease was prevented by insulin, dichloromethane, and ethyl acetate fractions, as well as subfractions F4, F5, and F5 sub-doses (*P* > 0.05 vs. nondiabetic vehicle group) (Figure 2(c)). The hind paws of diabetic control animals were more affected than the forepaws (∼3-fold decrease in performance score, *P*=0.0009) (Figure 2(d)). Changes in hindpaw performance score were mitigated by treatment with insulin, dichloromethane, and ethyl acetate fractions (*P*=0.016, *P*=0.04, and *P*=0.0013 vs. diabetic control group, respectively) (Figure 2(c)), as well as F4, F5 (*P*=0.036, *P*=0.014, and *P*=0.001 vs. diabetic control group, respectively) (Figure 2(d)) and F5 subdoses equal or higher than 0.34 mg/kg (*P* < 0.001 vs. diabetic control group) (Figure 2(d)). However, only the F5 dose 1.71 mg/kg preserved the hindpaw performance score at nondiabetic vehicle group values and better than insulin (*P*=0.028 vs. insulin group) (Figure 2(d)). The ability of F5 dose 1.71 mg/kg to preserve motor functions in STZ-induced diabetic mice were investigated further in other behavioral tests assessing cognitive and motor functions, namely, the OFT and the HBT tests.

### 3.4. Cognitive and Motor Function Indicators in the OFT and HBT Tests

**Figure 3:**
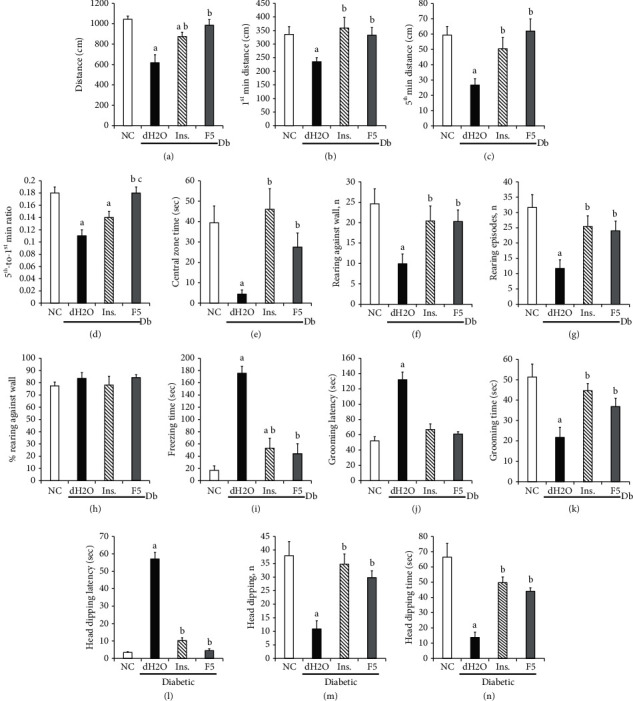
Open-field and hole-board tests. Effects of *G. kola* sub-fraction F5 (dose 1.71 mg/kg) on cognitive and motor indicators in the OFT (a–k) and HBT (l–n): total distance covered in the open-field arena (a), distances covered in the first (b) and in the fifth (last) (c) minutes, fifth-to-first minute ratio (d), total time spent in the field's central zone (e), rearing against wall (f) and total rearing episode number (g), rearing episode relative number (h), freezing time (i), grooming latency (j), and grooming time (k), head dipping latency in the hole-board apparatus (l), head dipping number (m) and total time (n). Note that treatment with insulin and F5 mitigated the changes observed in diabetic control animals (DC). ANOVA + LSD test: ^a^*P* < 0.05 vs. nondiabetic control (NC) group; ^b^*P* < 0.05 vs. DC group; ^c^*P* < 0.05 vs. insulin group. Data are mean ± SEM. *N* = 6 for NC and DC groups. *N* = 5 for insulin and test groups.

The results of the OFT and HBT tests are shown in Figure 3. In both tests, except for the relative number of rearing against the wall (total rearing against the wall episodes expressed as percent of total rearing episodes) in the open-field arena that was unchanged, all parameters were significantly altered in the diabetic control group compared to the nondiabetic vehicle group (Figure 3). More specifically, in the OFT test, the total distance traveled in the arena (*P*=0.001) (Figure 3(a)), distances traveled in the first and last minutes (*P*=0.018 and *P*=0.0006, respectively) (Figures 3(b) and 3(c)), the last-to-first minute distance ratio (*P*=0.008) (Figure 3(d)), the central zone time (*P*=0.005) (Figure 3(e)), rearing against the wall episodes (*P*=0.007) (Figures 3(f) and 3(h)), total rearing episodes (*P*=0.003) (Figure 3(g)), and the grooming time (*P*=0.009) (Figure 3(k)) were decreased, while the freezing episode time (*P*=0.002) (Figure 3(i)) and the latency to the first grooming episode (*P*=0.039) (Figure 3(j)) were increased in diabetic control group compared to the nondiabetic vehicle group. In the HBT test, the latency to the first head dipping was increased (*P*=0.012) (Figure 3(l)), while the number and time of head dipping episodes were decreased (*P*=0.001 and *P*=0.0007, respectively) (Figures 3(m) and 3(n)) in the diabetic control group compared to the nondiabetic vehicle group (Figure 3). These alterations of motor and cognitive functions' indicators were not observed in diabetic animals treated with F5 dose 1.71 mg/kg (*P* < 0.05 vs. diabetic control group), whose performances in OFT and HBT tests were comparable to the nondiabetic vehicle group (*P* > 0.05) (Figures 3(a)–3(n)). Except for the freezing time and the last-to-first minute distance ratio in the OFT test that were altered, insulin mitigated the changes in most of the HBT and OFT indicators (*P* < 0.05 to *P* < 0.001 vs. diabetic control group). Insulin failed to maintain the total distance traveled in the open-field arena, the last-to-first minute distance ratio, and the freezing time in the OFT test at normal values (*P*=0.012, *P*=0.039, and *P*=0.025 vs. nondiabetic vehicle group, respectively) (Figures 3(a)–3(n)).

### 3.5. Brain Histopathological Observations and Immunolabeling

**Figure 4:**
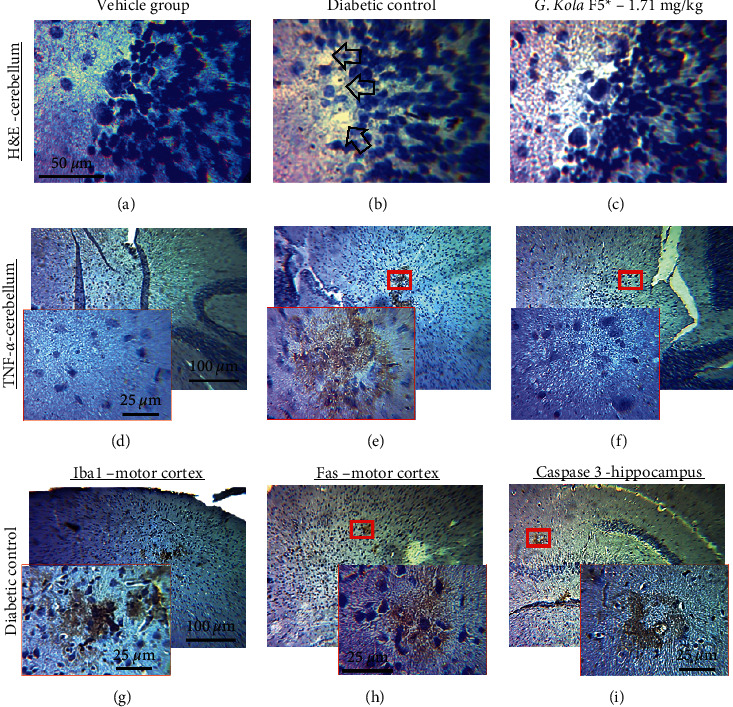
Histopathological and immunohistochemical observations. (a–c). H&E stained cerebellar cortex of representative cases of diabetic control, diabetic animals treated with (g) kola extract F5, and nondiabetic animals administered with vehicle solution. Note the missing (dead) Purkinje cells in the diabetic control indicated by the black arrows (b), unlike their nondiabetic (a) or (g) kola-treated (c) counterparts, where these neurons are still present (red arrows). Cases of nondiabetic animals or diabetic treated with (g) kola or insulin are not shown because they also displayed negative expressions of markers. D-F. Immunohistochemical expressions of TNF-*α* in deep cerebellar nuclei of representative cases of diabetic control (d), diabetic animal treated with *G. kola* extract F5 at dose 1.71 mg/kg (e), and nondiabetic animal (f). Insets represent magnifications of the red rectangle areas. Note the marked expression of TNF-*α* in the diabetic control and the absence of expression in the animal treated with *G. kola* (e) and in the control animal (F). G-I. Representative cases of diabetic controls showing the immunohistochemical expressions of iba-1 (g) and Fas receptor (h) in the motor cortex, and caspase 3 in the hippocampus (i). Note the marked expressions of all these markers.

Brain tissue observation (H&E staining) in diabetic control animals revealed perivascular edema, pericellular vacuolation, and signs of apoptosis (neuronal cell vacuolation and shrinkage), and central chromatolysis, particularly in large neurons like Purkinje cells, cerebellar deep nuclei neurons, and cortical pyramidal cells. Large neuron loss was also observed. Purkinje cell loss in the cerebellum of a representative diabetic control animal is shown in Figure 4(b). Large neuron loss and most of the aforementioned histopathological signs were not observed in diabetic animals treated with F5 at dose 1.71 mg/kg (Figure 4(c)).

Immunohistochemical labeling of neuronal cell death markers caspase 3 and Fas on brain sections confirmed these observations (Figures 4(h) and 4(i)). Notably, in diabetic control animals, Fas and caspase 3 were overexpressed in foci in the somatosensory and pyriform cortices, the motor cortex (Figure 4(h)), hippocampal formation (Figure 4(h) inset), septal nuclei, lateral hypothalamic area, medial amygdala nucleus, and cerebellar layers and deep nuclei. In addition, immunohistochemical labeling of the markers of inflammation TNF-*α* and iba1 in the brains of diabetic control animals revealed overexpression of these markers in the same brain structures, also in foci.  Figure 4(e) (respectively, Figure 4(g)) shows some inflammatory foci detected by anti-TNF-*α* (respectively, anti-iba1) in the deep cerebellar nuclei (respectively, in the motor cortex) of a representative diabetic control animal. Markers of neuronal death and neuroinflammation were not expressed in the brains of animals treated with F5 dose 1.71 mg/kg (data not shown).

### 3.6. Phytoconstituent Analysis of *G. kola* Sub-fraction F5

**Figure 5:**
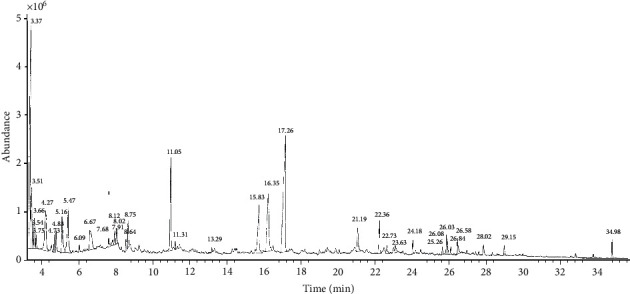
GC-MS chromatogram of fraction F5 of *G. kola*. Note the peaks of the most abundant compounds at 3.37 min (*N*,*N*-Dimethylethanolamine), 16.35 min (Isoniacinamide), and 17.26 min (*N*-Ethyl-2-carbethoxyazetidine).

GC-MS chromatogram of ethyl acetate subfraction F5 of *G. kola* extract is shown in Figure 5, and the phytoconstituents detected are listed in Table 2. The most phytoconstituents were the molecules of *β*-lactam family *N*-ethyl-2-carbethoxyazetidine (17.8%), *N*,*N*-dimethylethanolamine (15%), and isoniacinamide (9%) (Table 2). Their retention times were 17.26, 3.37, and 16.35 min, respectively (Figure 5). Less abundant phytoconstituents included 1-methoxy-1-methyl-1-silacyclohexane (6.7%), 4-methylproline methyl ester (5.3%), ethanedioic acid dimethyl ester (5%), 2(5H)-furanone (4.7%), 2-amino-4-methylbenzoic acid (3.2%), and 2-ethoxyethanol (2.3%) (Table 2). Flavoring agents were also detected, including 3-(methylthio)-2-butanone (3.8%) and butanedioic acid, hydroxy-, diethyl ester, (.+/−.)- (0.2%).

## 4. Discussion

Our results suggest that ethyl acetate subfraction F5 of *G. kola* methanolic extract is a potential candidate therapeutic for preventing the development of central nervous system complications of T1D. In this study, diabetic control animals displayed marked decreases in body weight and increases in blood sugar level, with motor signs such as significant decreases in the number of efficient steps without tremor (good steps) in the HGT, as well as decreases in time spent on the horizontal grid, possibly due to muscle fatigue associated with the systemic disease [40, 41]. Other authors reported similar observations in STZ-induced diabetic rodents [42, 43].

Except for water fraction, all fractions of *G. kola* extract tested improved the blood sugar level of diabetic animals and prevented T1D-mediated impairment of motor skill efficiency, i.e., the loss of fine motor skill efficiency. Notably, treatment with *G. kola* ethyl acetate fraction and insulin improved the body weight of diabetic animals beyond baseline values. Subfractions F2–F5 of ethyl acetate also improved the animal condition, nervous system dysfunction signs, and performance on the HGT of diabetic animals. Subfraction F5, which had the strongest effects, improved all factors measured in diabetic animals and restored the body weight to values comparable to the nondiabetic vehicle group, as in studies performed with crude *G. kola* and extracts [24, 25] and in models of neurodegenerative diseases and conditions [26, 44–47], indicating that F5 contains at least some major neuroprotective constituents of *G. kola*. Furthermore, F5 effects were dose dependent. Interestingly, at dose 1.71 mg/kg, F5-treated diabetic animals displayed appearance, behavior, and fine motor skills' efficiency comparable to the nondiabetic vehicle group animals, unlike insulin-treated animals. The HBT and OFT ethological tests also suggested that F5 subdose 1.71 mg/kg had stronger beneficial neuroactive actions than insulin. Notably, F5 treatment at this dose preserved all cognitive and motor indicators of diabetic animals to the nondiabetic vehicle group values, confirming the presence of strong neuroprotective molecules in that subfraction. Moreover, as suggested by low expressions of TNF-*α*, iba1, Fas, and caspase 3 in brain tissue, treatment with F5 also prevented neuroinflammation and neuronal loss, which are key mechanistic features of diabetic encephalopathy [15, 24, 25, 48].

Interestingly, the ability of almost all the fractions of *G. kola* extract to mitigate neuroinflammation confirmed the therapeutic potential of *G. kola* seeds in a T1D-like context [18, 24, 25] but indicated as well that *G. kola* seeds may induce their antidiabetic and neuroprotective activities through many constituents with adjuvant activities occurring as complex mixtures, as observed with various other medicinal plants [49–51]. Also supporting this hypothesis, GC-MS chromatography revealed that 34 secondary metabolites over 36 discovered accounted for 67.2% of the total content of sub-fraction F5, including flavonoids such as 2(5H)-Furanone (4.7%), 3-(methylthio)-2-butanone (3.8%), 1,3,6-trimethyl-2,4 (1H, 3H) -pyrimidinedione (0.8%), and 1,13-tetradecadien-3-one (0.5%). The neuroprotective activities of *G. kola* flavonoids were reported [31, 52, 53]. Thus, some of these compounds may contribute to the neuroprotective properties of F5. The most expressed phytoconstituents in F5 were *β*-lactam compounds *N*-ethyl-2-carbethoxyazetidine (17.8%) and *N*,*N*-dimethylethanolamine (15%). Beyond their well-established antimicrobial properties, *β*-lactam compounds may participate in the extensively reported anti-inflammatory and antidiabetic activities of *G. kola* seeds [21–23] and some *β*-lactam compounds were reported to show antiparkinsonian and hypoglycemic activities [54–56]. Notably, dimethylethanolamine (DMEA), a close molecular relative to *N*,*N*-dimethylethanolamine, is an established cholinergic antidepressive agent with therapeutic properties against dementia, dyskinesia, and epilepsy [57, 58]. Interestingly, the neuroactivity profiles of *N*,*N*-dimethylethanolamine and *N*-ethyl-2-carboxyazetidine are unknown. Thus, future studies addressing the therapeutic potential of these secondary metabolites may reveal novel and potent bioactive molecules useful against encephalopathy and other microvascular complications in T1D and related diseases.

The determination of the doses of fractions to be used is a limitation in this study. As already mentioned, our previous studies suggested that the effective dose of the crude extract for neuroprotection in STZ-induced diabetic rats and mice is approximately 100 mg/kg [24, 25]. Considering that the extraction and separation procedures could remove various minor constituents with adjuvant activities [49–51], mice were treated with doses of fractions equivalent to twice the content in 100 mg/kg of crude extract and doses of subfractions including approximately 10 times the amounts in dose 100 mg/kg of crude extract (6.85 mg/kg for F5) to maximize the chances to obtain strong biological responses in studies involving only a small number of animals. Notably, as animals treated with F5 at the 6.85 mg/kg dose showed potential signs of toxicity, including aggressiveness, we tested lower doses and observed fewer signs of toxicity and stronger neuroprotective activity at the dose of 1.71 mg/kg (1/4 dilution of the previous dose of F5). Moreover, the present study only assessed general neurotoxicity signs in diabetic animals. Thus, toxicity and dose-response studies should be performed in future studies to provide convincing data ensuring preclinical safety and providing an estimate of the therapeutic range for neuroprotective activity in a T1D-like context, given the importance for clinical drug development.

## 5. Conclusions

*G. kola* fractions mitigated or prevented the development of motor signs in diabetic animals and improved the body weight and blood sugar level. Motor impairment prevention-guided fractionation of *G. kola* in an STZ-induced mouse model of diabetic encephalopathy revealed the strongest effects in the ethyl acetate fraction and subsequently, in its fifth chromatography eluate (termed sub-fraction F5 in this study). F5 displayed dose-dependent effects in diabetic mice that were stronger than insulin treatment. GC-MS chromatography revealed that the *β*-lactam compounds *N*-ethyl-2-carbethoxyazetidine, *N*,*N*-dimethylethanolamine, and isoniacinamide were the dominant phytoconstituents in F5. The well-established neuroprotective properties of compounds of the dimethylethanolamine family may account at least partly for *G. kola*-induced cognitive function improvement. Future studies should assess the antidiabetic and neuroprotective properties of *N*,*N*-dimethylethanolamine, *N*-ethyl-2-carbethoxyazetidine, and isoniacinamide, which remain to be elucidated, considering the potential for developing a novel class of antidiabetic drugs able to prevent the development of central nervous system complications in particular, and microvascular complications in general.

## Figures and Tables

**Table 1:** Body weight change in STZ-induced diabetic mice (% weight at arrival), two weeks before, and two weeks after treatment with *G. kola* fractions.

	No treatment		Treatment	
	Week 1 post-STZ	Week 2 post-STZ	Week 3 post-STZ	Week 4 post-STZ
Non-diabetic control	12.9 ± 3.1	16.2	22.5 ± 2.4	30.9 ± 4.2
Diabetic control	−13.6 ± 1.1^a^	−18.8 ± 1^a^	−28.2 ± 1.1^a^	−25.7 ± 2.9^a^
DM + Insulin	−15.1 ± 2.8^a^	−22.4 ± 2.1^a^	0.6 ± 3.1^a,b^	3.8 ± 2.2^a,b^
DM + *G. Kola* methanolic extract fractions				
Water	−10.4 ± 2.5^a^	−17.6 ± 1.2^a^	−31.9 ± 1.3^a,b^	−30.1 ± 4.4^a,b,c^
Hexane	−9.4 ± 1.6^a^	−19.7 ± 2.8^a^	−7.5 ± 1.5^a,b,c^	−7.8 ± 3.6^a,b,c^
Dichloromethane	−17 ± 4.7^a^	−23.2 ± 3.5^a^	−12.6 ± 0.8^a,b,c^	−11.4 ± 3^a,b,c^
Ethyl acetate	−6.3 ± 4.1^a^	−24.7 ± 3.3^a^	−6.4 ± 3.9^a,b^	1.7 ± 2.7^a,b^
DM + ethyl acetate sub-fractions				
Sub-fraction F2	−11.2 ± 4.2^a^	−16.8 ± 5.66^a^	−22.9 ± 1.8^a,b,c^	−21 ± 3.8^a,b,c^
Sub-fraction F3	−10.8 ± 2.7^a^	−22.2 ± 2.1^a^	−30 ± 2.5^a,c^	−31.9 ± 1.5^a,c^
Sub-fraction F4	−15.4 ± 2.7^a^	−22.7 ± 2^a^	−11.4 ± 1.2^a,b,c^	−11.9 ± 3.1^a,c^
Sub-fraction F5	−12.2 ± 2.3^a^	−18.4 ± 4.2^a^	9 ± 4^a,b^	27.4 ± 6.9^b,c^
DM + Sub-doses of ethyl acetate sub-fraction F5				
0.34 mg/kg	−18.6 ± 4.1^a^	−32.7 ± 3^a^	−15.1 ± 4^a,b,c^	−13.9 ± 2^a,b,c^
0.69 mg/kg	−14.2 ± 3.3^a^	−17.3 ± 3^a^	0.9 ± 3.7^a,b^	−0.04 ± 4.9^a,b^
1.71 mg/kg	−17.3 ± 2.7^a,b^	−27.7 ± 1.8^a,^	1.3 ± 4.6^a,b^	3.1 ± 8.4^a,b^
3.43 mg/kg	−19 ± 3.3^a,b^	−19.1 ± 1.1^a^	4.6 ± 3.5^a,b^	14.6 ± 1.7^a,b,c^

ANOVA + LSD test: ^a^*P* < 0.05 vs. nondiabetic control group; ^b^*P* < 0.05 vs. diabetic control; ^c^*P* < 0.05 vs. insulin group. Data are mean ± SEM. *N* = 7 for healthy and diabetic controls. *N* = 5 for insulin and other groups.

**Table 2:** Constituents of *G. kola* sub-fraction F5 revealed by GC-MS chromatography.

Phytoconstituents	RT (min)	Area (%)	Mol. W (amu)	Cas number	PubChem CID
*N*-Ethyl-2-carbethoxyazetidine	17.26	17.8	157.11	054773-05-6	558346
*N*,*N*-Dimethylethanolamine (DMEA)	3.37	15	89.08	000108-01-0	7497
Isoniacinamide	16.35	9.0	122.05	000098-92-0	15074
1-Methoxy-1-methyl-1-silacyclohexane	11.05	6.7	204.06	056196-50-0	582107
4-Methylproline methyl ester	15.83	5.3	143.06	054571-66-3	45089984
Ethanedioic acid dimethyl ester	4.27	5	118.03	000553-90-2	11120
2(5H)-furanone	5.47	4.7	84.02	000497-23-4	10341
3-(Methylthio)-2-butanone	3.54	3.8	118.05	053475-15-3	103788
2-Dimethylsilyloxypentane	6.67	3.5	146.11	053691-19-3	6329237
2-Amino-4-methylbenzoic acid	5.16	3.2	151.06	1000222-86-6	75316
(1R)-(-)-thiocamphor	22.36	2.4	168.1	053402-10-1	1714210
2-Ethoxyethanol	3.66	2.3	90.07	000110-80-5	8076
2-Benzyloxyphenylacetonitrile	21.19	2.2	223.1	1000318-85-3	561222
Diethoxymethane	8.03	1.9	104.08	000462-95-3	10024
Diethoxydimethoxysilane	4.84	1.5	222.14	053172-91-1	519523
*n*-Hexadecanoic acid (palmitic acid)	26.58	1.6	256.24	000057-10-3	985
Propanoic acid, pentamethyldisilanyl ester	8.75	1.5	204.1	017728-88-0	554466
3-Propylphenol	3.54	1	136.1	057736-55-7	69302
Hexadecanoic acid, methyl ester	26.01	1.1	270.26	000112-39-0	8181
1,2-Benzenedicarboxylic acid, diisooctyl ester	34.98	0.9	390.28	027554-26-3	33934
2,3-Dimethylpentanal	4.73	0.9	114.1	032749-94-3	61917
2-(Trifluoromethyl)-10h-phenothiazine	28.02	0.8	267.03	001478-61-1	7082
2-Fluoro-2-methylpropane	7.69	0.7	76.07	000353-61-7	9626
Hexamethylcyclotrisiloxane	8.65	0.8	222.06	000541-05-9	10914
Cyclohexanecarboxylic acid, decyl ester	23.24	0.8	228.21	000544-63-8	582158
1,3,6-Trimethyl-2,4(1H,3H)-pyrimidinedione	24.18	0.8	154.07	000500-99-2	26075
1-Methyl-3-piperidinemethanol	22.77	0.7	129.12	007583-53-1	97998
2-Amino-5-nitro-phenol	26.08	0.7	409.36	1000327-78-3	4984721
Methyloctadecyldiethoxysilane	3.75	0.6	386.36	067859-75-0	105838
*N*-Methoxy-*N*-methylacetamide	7.92	0.5	103.06	078191-00-1	2734716
1,13-Tetradecadien-3-one	26.24	0.5	208.18	058879-40-6	337818
Octadecanoic acid, methyl ester	29.15	0.5	298.29	000112-61-8	110444
1-Anthracenamine	6.09	0.4	193.09	000610-49-1	11885
*N*,*N*-dimethyl-1,3-propanediamine	11.31	0.3	102.12	000109-55-7	7993
4-Methyl-2,7-dioxa-tricyclo [4.4.0.0(3,8)] decane	25.79	0.4	154.1	1000193-48-4	620310
Butanedioic acid, hydroxy-, diethyl ester, (.+/−.)-	13.29	0.2	190.08	000626-11-9	24197

AMU: atomic mass units. Mol. W: molecular weight. RT: Retention time.

## Data Availability

Data will be made available upon request or submitted if possible to the journal website.

## Abbreviations

DAB: 3,3′-diaminobenzidine hydrochloride
H&E: Hematoxylin and eosin staining
HBT: Hole-board test
HGT: Horizontal grid test
i.p.: Intraperitoneal injection
LSD: Least significant difference
OFT: Open-field test
p.o.: *Per os*
STZ: Streptozotocin
TNF-*α*: Tumor necrosis factor-alpha
T1D: Type 1 diabetes mellitus.
